# A New Strategic Approach to Successful Aging and Healthy Aging

**DOI:** 10.3390/geriatrics3040086

**Published:** 2018-11-29

**Authors:** Roger Y. Wong

**Affiliations:** Division of Geriatric Medicine, Department of Medicine, Faculty of Medicine, University of British Columbia, Vancouver, BC V5Z 1M9, Canada; roger.wong@ubc.ca

Successful aging is not a new concept, although its definition remains controversial, because of its multi-dimensional nature. To the biomedical scientist, successful aging is defined by the absence of disease, physical, and cognitive disability [1]. This is distinct from usual aging, which is associated with age-related decline in physical and cognitive function. To the social scientist, successful aging emphasizes life satisfaction and personal well-being, usually achieved through socialization [2]. 

Healthy aging is a related concept in that it captures the essence of physical and cognitive functional preservation, but without the requirement of disease avoidance [3]. Healthy aging is therefore a more inclusive concept, one that more accurately describes more individuals as the population ages. The World Health Organization (WHO) defines healthy aging as the process of developing and maintaining the functional ability that enables well-being in older age [4]. Under the WHO conceptual construct, there are four essential requirements for healthy aging: (i) a change in the way we think about aging and older people, (ii) creation of age-friendly environments, (iii) alignment of health systems to the needs of older people, and (iv) development of systems for long-term care [5]. These four requirements have clinical, research, and policy implications [6,7], therefore lending themselves to form the four pillars of a new strategic approach to healthy aging. 

To change the way we think about aging, a fundamental pre-requisite is to revisit the theoretical constructs that we use to study aging. Historically, the focus has been on single-organ diseases. The emergence of geroscience is one way of transforming our thinking about older people. Geroscience refers to an inter-disciplinary approach to understanding the genetic, molecular, and cellular mechanisms that make aging a risk factor and driver of chronic health conditions in older people [8]. A number of the world-leading aging research institutions have already adopted this innovative approach, in contrast to the traditional single-organ or disease-specific approach. Translational research studies in geroscience have begun to reveal new insights in a number of areas [9], including genomics (such as the relationship between the Apolipoprotein E allelic composition and longevity), proteomics (such as the role of cytokines in cerebrovascular disease and Alzheimer’s disease), metabolomics (such as the presence of abnormal metabolites in diabetes mellitus and certain cancers), and microbiomics (such as the impact of losing microbiome diversity with increased bacilli and proteobacteria in older people). Geroscience is a new and different strategic approach towards advancing our understanding about healthy aging. Innovative health research and educational platforms should be developed to embrace and support geroscience as an emerging field. 

The environment is an important determinant of health, and the creation of an age-friendly environment has attracted significant attention in recent years, both in the scientific community and society at large. The WHO characterizes these environments as accessible, equitable, inclusive, safe and secure, and supportive [10]. There are macro and micro examples of age-friendly environments. At the macro level, global cities are classified as age-friendly if they perform well under a list of pre-specified core indicators [11]. The indicators are in turn organized within a program logic model, with inputs, outputs, outcomes, and impact. At the micro level, personal living spaces can become age-friendly with deliberate considerations to implement the same WHO characteristics as described earlier. An important enabler is technology. Innovative examples include the Internet of Things, wearable devices, tablet devices that are community-based and connected to electronic health records, social robots, various forms of decision support, and machine learning, to name a few. Recent efforts have also explored the combination of age-friendly environments and effective healthcare delivery. The patient medical home (PMH) is a good example that integrates primary care and community needs [12]. The attributes of the PMH are evidence-based to provide care that is person-centred care, timely, comprehensive, continuous, coordinated, team-based, and subject to quality improvement. Overall, the creation of age-friendly environments provides an essential foundation for healthy aging. A natural corollary is the need to assemble inter-disciplinary research and implementation teams that bring together expertise from medicine, engineering, architecture, business, and sociology. 

The appropriate alignment of health systems to the needs of older people plays a pivotal role in supporting healthy aging. Many jurisdictions around the world have come a long way in transforming health systems to be more age-friendly, as evidenced in the significant advances and improvements in hospital medicine for older people [13]. However, transformative changes of this nature are often opportunistic, inconsistent, and not widespread [14]. Moreover, gaps in healthcare delivery continue to exist, especially with respect to primary care delivery and at transition points, such as acute hospital care and long-term care [15]. Moving forward, age-friendly health systems should focus on healthy aging and preventive geriatrics. The programmatic emphasis should be on primary care geriatrics. While innovation may be disruptive in nature, future health systems should augment connectivity (actual and virtual) between community care, acute care, and long-term care. A system-based approach of quality improvement can be helpful [16]. The process of health systems realignment will take time. Proactive and meaningful engagement of older people throughout the process is crucial for success. In addition, there is an opportunity to better integrate health service delivery and research, preferably starting at the point of entry into health systems. 

Last but not least, the development of systems for long-term care is important for healthy aging. Innovative models of long-term care are needed to support aging in place, which refers to the ability to live in a person’s own community safely, independently, and comfortably, regardless of age, income, or ability level [17]. This is the aspiration and desire of older people who are aging healthily. Long-term care system improvements should therefore address how customized services can be deployed in the community when individual health needs change, often as a result of physical and/or cognitive functional decline. Effective interventions are likely multi-component in nature and require inter-professional delivery, although it might be difficult to delineate the exact benefit conferred by each individual intervention component [18]. Data science can be a helpful enabler. Health informatics (“big data”) can incorporate longitudinal personal health data from population-based electronic health records to predict important health outcomes. The abundance of population-based physiologic data would also allow deep learning or machine learning to take place, so that each older person can be risk-stratified in terms of the person’s own level of frailty and risks of developing various adverse events, such as the common geriatric issues of delirium and falls. 

This article highlights the four pillars of a new strategic approach to healthy aging. To ensure its successful implementation, stakeholder education is the key [19]. This includes education for health professionals, researchers, policy decision makers, and most importantly, the general public. A recent study suggests that the perception of aging in the community can influence the effect of healthy aging programs [20]. Education can play an important role in empowering people and altering perception. 

Healthy aging is important for all of us and it touches every one of us. Act before it is too late. 
